# Cognitive biases as Bayesian probability weighting in context

**DOI:** 10.3389/fpsyg.2025.1572168

**Published:** 2025-08-06

**Authors:** Bruno Kopp

**Affiliations:** Cognitive Neuropsychology, Department of Neurology, Hannover Medical School, Hannover, Germany

**Keywords:** Bayesian inference, decision making, cognitive biases, learning from context, computational models, decision support

## Abstract

**Introduction:**

Humans often exhibit systematic biases in judgments under uncertainty, such as conservatism bias and base-rate neglect. This study investigates the context dependence of these biases within a Bayesian framework.

**Methods:**

Forty-eight participants made subjective probability judgments in 12 scenarios requiring the integration of prior probabilities and evidence likelihoods.

**Results:**

Results show that task context mediates the weighting of priors and evidence: small-world scenarios (e.g., urn problems) emphasize priors, thus amplifying the conservatism bias, whereas large-world scenarios (e.g., taxi problems) increase sensitivity to evidence, leading to base-rate neglect. Presenting probabilistic information as relative frequencies rather than probabilities did not attenuate these biases.

**Discussion:**

To explain these findings, we propose the Adaptive Bayesian Cognition (ABC) model, which describes how individuals dynamically adjust the weighting of priors and evidence. By integrating normative Bayesian principles with psychological insights, the ABC model recasts cognitive biases as adaptive strategies shaped by capacity constraints and meta-learning in specific contexts. These findings bridge cognitive psychology, behavioral economics, and computational modeling to provide a unified framework for understanding subjective probability, probability weighting, and decision making under uncertainty. This work also informs the design of decision support systems.

## Introduction

The experimental demonstration of cognitive biases, particularly narrower probabilistic biases such as conservatism bias and base-rate neglect, has been taken as evidence that human probabilistic reasoning deviates in significant ways from normative Bayesian theory (Kahneman et al., 1982). These two probabilistic biases have led to two different prevailing interpretations: In the case of conservatism bias, where individuals inadequately update their prior beliefs in response to additional evidence, it is often argued that these inherently Bayesian cognitive processes are systematically biased in some way, perhaps due to certain cognitive limitations (Phillips and Edwards, 1966; Peterson and Beach, 1967). In contrast, base-rate neglect—the subsumed tendency to underweight prior probabilities (or base rates) when evaluating novel evidence—has often been taken as evidence that human cognition is fundamentally non-Bayesian in nature, implying that intuitive cognition operates via heuristic processes (Gigerenzer and Brighton, 2009; Brighton and Gigerenzer, 2015) rather than probabilistic computation (Kahneman and Tversky, 1973). This research has led to the conclusion that at least some aspects of human reasoning appear to be boundedly rational or even non-rational (Kahneman, 2003; Barbey and Sloman, 2007), as Bayesian theory has often been equated with rationality. This research has therefore threatened the validity of Bayesian models as descriptive of human cognition (Gigerenzer and Murray, 1987; Anderson, 1990; Oaksford and Chater, 2007; Griffiths et al., 2010, 2024).

The current study focuses on understanding the context sensitivity of these probabilistic biases. First, I examine how task content influences the weighting of prior information and evidence in belief updating. Traditionally, conservatism bias has been studied in so-called small-world (Savage, 1954) tasks, such as abstract urn tasks (Phillips and Edwards, 1966), with their statistical look and feel of randomness, which may make prior probabilities highly salient. In contrast, base-rate neglect has often been studied in realistic large-world (Savage, 1954) scenarios, such as the well-known cab problem (Kahneman and Tversky, 1972), where there is an impression of potentially causal variables and seemingly convincing eyewitness evidence (Tversky and Kahneman, 1977, 1980).

However, typical small-world and large-world tasks share a common structure: they present prior information and evidence, and require participants to update their beliefs in order to report subjective posterior probabilities. Importantly, the relative emphasis on prior information vs. evidence is not fixed, but can be dynamically adjusted as a function of task characteristics (Gigerenzer et al., 1988; Koehler, 1996). In particular, while the role of prior probabilities may be emphasized in urn tasks, the emphasis in realistic scenarios may shift to evidence due to its often salient nature. It is immediately apparent that when priors dominate, subjective probability judgments may reflect conservatism and underweight the impact of evidence, whereas when evidence is prioritized, this source of information may be over-relied upon, possibly at the expense of priors.

Second, task context often implicitly influences the diagnosticity of evidence by varying the amount of evidence provided. In typical small-world tasks, multiple samples of evidence are presented. This procedural detail increases the normative impact of evidence on posterior beliefs. In addition, repeated sampling clearly reinforces the random nature of the problem, and facilitates a more deliberate weighting of prior beliefs. In contrast, single instances of evidence are typically presented in realistic, large-world scenarios such as the cab problem. The limited presentation of evidence may lead individuals to rely more heavily on causal interpretation tendencies (Tversky and Kahneman, 1977, 1980). This, in turn, may reinforce seemingly non-Bayesian strategies such as base-rate neglect.

Third, another important factor is the format in which intuitive judgments are framed, specifically whether information is presented in terms of the probability of a single event or in terms of its relative frequency in a long series of events. Building on previous research (Gigerenzer et al., 1988; Sedlmeier and Gigerenzer, 2001), frequency framing is expected to favor probabilistic computation, whereas probability framing is expected to be more closely aligned with heuristic processing, potentially reinforcing seemingly non-Bayesian strategies, including the neglect of base rates.

The current study examines the context sensitivity of probabilistic biases by systematically manipulating three key variables: task content, contrasting small-world (urn) with realistic large-world (taxi) scenarios; amount of evidence, contrasting single-event with multiple-event evidence conditions; and task framing, contrasting frequency with probability framing. The core idea is that cognitive attention mediates the dynamic, putatively competitive integration of priors and evidence, with task context shaping the influence of each, potentially allowing contextual adaptation to optimize decision outcomes.

Based on these considerations, the prediction can be derived that in large-world scenarios, such as the cab problem, cognitive attention is biased toward evidence, leading to base-rate neglect and sensitivity to the amount of evidence. In contrast, in small-world tasks, such as the urn problem, priors are prioritized, leading to conservatism and reduced sensitivity to evidence samples, so that discrimination between single and multiple evidence conditions is limited. In the current study, with its manipulation of task content, amount of evidence, and framing, this analysis predicts interactive effects of content and evidence on subjective probability judgments, with the effect of the evidence manipulation being stronger in large-world than in small-world tasks. According to previous work, frequency framing should reduce both probabilistic biases across task content and evidence conditions by facilitating probabilistic computation.

## Methods

### General remark

The data reported in this study were collected in May 1988 as part of an unpublished diploma thesis at the University of Konstanz (Kopp, 1988; supervisors: G. Gigerenzer, W. Hell). The study was designed to investigate cognitive processes relevant to human judgment under uncertainty. Data were collected in accordance with the ethical standards and research practices of the time, including obtaining informed consent from all participants. The written informed consent included personal information that was collected only in a paper-and-pencil format. The digitized study data on which the current re-analysis is based are anonymized and cannot be linked to an individual with reasonable effort. With the exception of an oral presentation at the Conference of Experimental Psychologists (Bamberg, Germany, March 21, 1989), the data have not been previously published.

### Participants

All participants were recruited at the University of Konstanz through local advertisements on bulletin boards. A total of 48 individuals participated (46 students, 2 staff members). There were 25 males and 23 females among the participants. The median age of the sample was 23 years, ranging from 18 to 39 years. Twenty-three students were enrolled in psychology and 23 in other disciplines (10 humanities, 8 social sciences, 5 natural sciences). Their median number of semesters was 2, ranging from 2 to 15 semesters. There were no other inclusion or exclusion criteria. Although some of the participants, especially the psychology and social science students, may have had formal training in statistics, Bayesian statistics was not part of their statistics training at the time. Three individuals indicated that they had heard of Bayes' theorem, but their knowledge was rudimentary. Participants received either course credit (relevant to the majority of psychology students) or monetary compensation (DM 7.50 per hour).

### Materials and measures

All participants received 12 probability judgment tasks, designed to examine the effects of three independent variables on human judgment under uncertainty. First, task content was manipulated so that the task set included paradigmatic small-world (urn tasks) and large-world (cab problems) scenarios in order to examine the potential content-dependency of cognitive processes relevant to human judgment under uncertainty.

Urn tasks are a paradigmatic Bayesian problem because they illustrate the core process of Bayesian inference: updating prior beliefs in light of evidence to derive a posterior probability. Bayesian inference has three components: prior probability, evidence (likelihood), and posterior probability. In the urn task, the prior probability is the initial belief about the probability of a selected urn being blue or green based on base rates, such as the fact that 85% of the urns in a set of urns are green and 15% are blue. This base rate represents the prior probability of selecting an urn of each color. The evidence comes from drawing balls from the selected urn with given probabilities (e.g., 80% blue balls in blue urns, 20% blue balls in green urns). This evidence provides additional information that must be integrated with the prior belief to update the probability estimate. The posterior probability is the updated belief about the selected urn after considering some evidence.

The posterior probability is calculated using Bayes' theorem (Equation 1), which combines the prior probability and the likelihood of the evidence. Mathematically, assuming a blue ball was drawn as evidence, the posterior probability that a blue urn was selected is


(1) $$\begin{array}{l} p\left(blue\;urn|blue\;ball\right)=\frac{p\left(blue\;urn\right)\times p\left(blue\;ball|blue\;urn\right)}{p\left(blue\;ball\right)} \end{array}$$

The nominator is the product of the prior probability and the evidence likelihood. The denominator, *p*(*blue ball*), accounts for all possibilities, including the blue ball being drawn from a green urn. This normalization ensures that the posterior probabilities sum to 1 and correctly reflect the updated belief. In the example above, by substitution, $\frac{p\left(blue\;urn\right)\times p\left(blue\;ball|blue\;urn\right)}{p\left(blue\;ball\right)}=\frac{0.15\times 0.80}{0.15\times 0.80+0.85\times 0.20}\approx 0.414$. Therefore, the Bayesian posterior probability is *p*(*blue urn*|*blue ball*)≈0.414. In other words, the evidence of drawing a blue ball induces an update in the belief that a blue urn was selected (from a priori 0.15 to posteriori 0.41), but despite the evidence of a blue ball, it remains slightly less likely that a blue urn was selected than a green urn under these particular circumstances.

All of the large-world scenarios used were structurally equivalent to small-world (urn) tasks. They were also equivalent to or derivatives of the original cab problem, which is documented below (Tversky and Kahneman, 1980):

“A Cab was involved in a hit and run accident last night. Two Cab companies, called Green and Blue operate in the City. You are given the following data:

- 85% of the Cabs are Green and 15% are Blue.
- A witness identifies the Cab as blue. The court tested the reliability of the witness under the circumstances that existed on the night of the accident and concluded that the witness could correctly identify each of the two colors 80% of the time but would fail 20% of the time.

What is the probability that the Cab involved in the accident was Blue rather than Green?”

The problem structure of the cab problem mirrors that of the small-world problem: base rates are provided by the relative frequencies of green and blue taxis (equivalent to urns), and the probability of witness testimony (equivalent to ball-drawing likelihoods) is provided by the reliability of witness testimony. However, despite the structural equivalence of small-world and large-world scenarios, probabilistic judgments in small-world and real-world scenarios often deviate in several content-dependent ways from rational (i.e., Bayesian) judgments because, as discussed above, they either neglect the base rate (base-rate neglect, typically observed in large-world problems) or fail to properly integrate the evidence (conservatism bias, typically observed in small-world problems). The combination of these scenarios is therefore an essential tool for studying these two probabilistic biases and for understanding the content-dependent limitations of Bayesian reasoning.

The task content actually comprised three levels: The first level of task content included the paradigmatic small-world scenario (an urn task), in which participants infer posterior probabilities based on the composition of a set of urns (yielding prior probabilities) and the composition of the balls in the urns (yielding evidence likelihoods). The next two levels of task content consisted of two large-world scenarios. The standard cab problem, in which the relative frequencies of green and blue taxis in the city reflect prior probabilities and witness reliability reflects evidence likelihood, was complemented by a modified version of the cab problem in which prior probability is made causally relevant by specifying the number of drivers involved in hit-and-run incidents rather than general taxi frequencies (Tversky and Kahneman, 1977, 1980).

Human decision making differs in small-world and large-world scenarios (Savage, 1954). In small-world problems, people rely on statistical reasoning characterized by well-defined probabilities (e.g., Gigerenzer et al., 1988). Here they operate in a “non-causal” framework, as in an “empire of chance.” In contrast, large-world problems involve ill-defined probabilities and often contain information that invites causal interpretations. Kahneman and Tversky (1973) showed that people prioritize causal relevance over base rates, while Koehler (1996) highlighted perceived causality as key to the application of prior probabilities in large-world problems. These findings emphasized a shift from statistical reasoning to causality-driven reasoning in large-world scenarios. The modified version of the cab problem, in which the prior probability was made causally relevant, was designed to test whether the perceived causal relevance of prior probabilities mediates base-rate neglect as it occurs in the standard version of the cab problem.

Evidence levels in judgment studies have been manipulated by presenting either a single piece of evidence, such as a ball drawn from an urn or a single eyewitness identification, or multiple pieces of evidence, such as three consecutive balls drawn from an urn (with replacement) or three independent eyewitness identifications. The biases of conservatism and base-rate neglect are closely related to this structure of empirical evidence. Conservatism typically involves cautious belief updating in response to multiple pieces of evidence in urn tasks, leading individuals to underweight new information relative to the Bayesian norm. In contrast, base-rate neglect (Kahneman and Tversky, 1973; Bar-Hillel, 1980) often occurs with single pieces of evidence in real-world scenarios, where individuals tend to overlook prior probabilities in favor of salient, case-specific details. These paradigms underscore the critical role that the content and amount of evidence play in shaping probabilistic reasoning, and unfortunately, these two factors are deeply confounded in the literature.

Third, the task framing was adjusted to present the problem as either a single-case problem, focusing on the probability of a particular event, or a long-term problem, emphasizing relative frequencies over repeated events. Previous work highlights the influence of this type of framing on reasoning, particularly the distinction between probabilities and frequencies (Sedlmeier and Gigerenzer, 2001). When probabilities were presented in abstract, single-event formats, people often struggled to reason correctly, presumably because this format is poorly aligned with intuitive cognitive processes. In contrast, frequency formats—expressing probabilities as frequencies over repeated events—were thought to be more consistent with how people naturally process information. The study showed that frequency framing facilitates Bayesian reasoning and reduces biases such as base-rate neglect. These findings suggest that framing probabilistic information as probabilities or frequencies has profound implications for human judgment under uncertainty.

These three independent variables created a factorial design with three levels of content, two levels of evidence, and two levels of framing, resulting in 12 different versions of a structurally identical Bayesian inference problem. This design allowed for the investigation of how these changes in content, amount of evidence, and framing affected probabilistic reasoning, particularly in terms of adherence to Bayesian principles. The exact wording of the 12 different versions is documented in Supplementary material A. Their construction was done by hand, and no software was used for this task.

Participants received the 12 task scenarios in a booklet. At the end of each task, participants were asked to report two posterior probabilities (on a percentage scale). For example, in the original cab problem, these probability judgments were prompted by the questions: (1) What is the probability that the taxi involved in the accident was blue? and (2) What is the probability that the taxi involved in the accident was green? The general task instructions emphasized that these two percentages should sum to 100.

### Procedure

Participants completed the booklet containing 12 scenarios, each requiring two probability judgments. After providing written informed consent, they were given task instructions before beginning the scenarios, which took ~45–90 min to complete. The study used a within-subjects design, with the order of scenarios randomized for each participant. To prevent participants from treating the scenarios as repetitive, the numerical values for prior probabilities and evidence likelihoods were systematically varied, as shown in Table 1. Each participant encountered each unique combination of numerical values exactly once across all scenarios. This approach required the construction of all 12 scenarios in all possible value combinations, resulting in 144 different scenario-value pairs. Each of the 12 unique numerical combinations per scenario was used four times across the sample of 48 participants. The average prior probability per scenario was 0.15, and the average evidence likelihood per scenario was 0.775, resulting in average Bayesian posteriors of 0.378 (*n* = 1) and 0.878 (*n* = 3).

**Table 1:** Bayesian posterior probabilities for the 12 unique numerical combinations of prior probabilities and evidence likelihoods, with three prior probabilities (0.1, 0.15, 0.2) and four evidence likelihoods (0.7, 0.75, 0.8, 0.85), separately for the two levels of evidence (*n* = 1, *n* = 3).

***n* = 1**	**0.7**	**0.75**	**0.8**	**0.85**
0.1	0.206	0.25	0.308	0.386
0.15	0.292	0.346	0.414	0.5
0.2	0.368	0.429	0.5	0.586
***n*** = **3**				
0.1	0.585	0.75	0.877	0.953
0.15	0.692	0.827	0.919	0.97
0.2	0.761	0.871	0.941	0.978

### Statistical analysis

JASP version 0.18.3.0 was used as the software for all data analyses. The primary statistical analyses used to test hypotheses were Bayesian repeated measures ANOVAs. The measures used for these hypothesis tests were log odds, defined as $\log 𝒪=\log\frac{p_{1}}{p_{2}}$ where *p*_1_ is the first (see definition above) subjective probability (i.e., provided percentage divided by 100) and *p*_2_ is the second (see definition above) subjective probability (i.e., provided percentage divided by 100), with the constraint that *p*_1_+*p*_2_ = 1 for each scenario. If the sum of these two probabilities did not equal 1, a correction was made by multiplying both probabilities by the scaling factor $\frac{1}{p_{1}+p_{2}}$. There were no dropouts. Regarding the handling of missing data, there was a single missing probability pair in one of the urn tasks from one participant, which was replaced by the average of all probability pairs from the remaining 47 participants in the same scenario.

JASP's power analysis module, based on jpower (Morey, 2021), was used to provide a rough indication of the statistical power of the analyses. Assuming a one-sample *t*-test rather than a Bayesian repeated measures ANOVA, one would need a sample size of 44 to be confident with a probability ≥0.9 of detecting an effect size of |δ| ≥0.5, assuming a two-tailed criterion that allows for a maximum Type I error rate of α = 0.05. One would need a sample size of 36 to be confident with a probability ≥0.9 of detecting an effect size of |δ| ≥ 0.5, assuming a one-tailed criterion that allows for a maximum Type I error rate of α = 0.05. Thus, the sample size of 48 can be considered sufficient to detect a moderate-sized effect with reasonable statistical power under standard types of analyses and assumptions.

## Results

Table 2 shows the averaged posterior probabilities (denoted *p*) and the corresponding log odds (denoted log𝒪), separately for all 12 scenarios constructed by combining task content (urn = urn task, cab s = standard cab problem, cab m = modified cab problem), level of evidence (*n* = 1, *n* = 3), and framing (probabilities, relative frequencies).

**Table 2:** Summary statistics (*N* = 48), separately for the 12 scenarios used, which were obtained by manipulating the task content (urn = urn task, cab s = standard cab problem, cab m = modified cab problem), the level of evidence (evidence_1_: *n* = 1, evidence_3_: *n* = 3), and the framing of problems [pro, probabilities; fre, (relative) frequencies].

	**p_Bayes_**	***M*** **_psubjective_**	**log𝒪_Bayes_**	***M*log𝒪**	**SD log𝒪**
urn_1_ pro	0.378	0.381	−0.498	−0.483	0.923
urn_3_ pro	0.878	0.363	1.976	−0.565	0.946
urn_1_ fre	0.378	0.435	−0.498	−0.259	0.948
urn_3_ fre	0.878	0.409	1.976	−0.367	0.951
cab s_1_ pro	0.378	0.601	−0.498	0.411	0.874
cab s_3_ pro	0.878	0.717	1.976	0.933	0.931
cab s_1_ fre	0.378	0.773	−0.498	1.228	0.831
cab s_3_ fre	0.878	0.840	1.976	1.659	0.928
cab m_1_ pro	0.378	0.653	−0.498	0.631	0.771
cab m_3_ pro	0.878	0.705	1.976	0.871	0.880
cab m_1_ fre	0.378	0.688	−0.498	0.792	0.764
cab m_3_ fre	0.878	0.835	1.976	1.621	0.894

All posterior probabilities mentioned here refer to the outcome with the lower prior probability. The normative Bayesian posterior probabilities, *p*_*Bayes*_, the mean subjective posterior probabilities, M *p*_*subjective*_, the Bayesian log odds (log𝒪_*Bayes*_), and the mean, M, and the standard deviation, SD, of the subjective log odds (log𝒪) are provided for each scenario (with an average prior probability of 0.15 and an average likelihood of evidence_1_ of 0.775).

As shown in Table 2, there are notable differences in the averaged subjective probability judgments based on task content, particularly between the urn and cab scenarios. In the evidence_1_ conditions, the average subjective posteriors in the urn_1_ tasks, ranging from 0.381 (urn_1_ pro) to 0.435 (urn_1_ fre), were well-calibrated to the Bayesian posterior (0.378). In contrast, the average subjective posteriors in the cab_1_ scenarios, ranging from 0.601 (cab s_1_ pro) to 0.773 (cab s_1_ fre), were strongly biased toward the likelihood of the evidence (0.775). This indicates poor calibration to the Bayesian posterior and is commonly referred to as base-rate neglect.

Examining sensitivity to evidence reveals that average subjective probabilities in the urn tasks are largely insensitive to evidence manipulations. In the urn_3_ tasks, the average subjective posteriors, ranging from 0.363 (urn_3_ pro) to 0.409 (urn_3_ fre), were more or less indistinguishable from those in the urn_1_ tasks, indicating a strong conservatism bias. Thus, these average subjective posteriors were poorly calibrated to the Bayesian posterior of 0.878. In contrast, average subjective probability judgments in the cab scenarios exhibited limited sensitivity to evidence. Subjective posteriors in cab_3_ scenarios (ranging from 0.705 in cab m_3_ pro to 0.840 in cab s_3_ fre) more closely matched the Bayesian posterior.

Tasks presented in a frequency format generally elicited slightly higher average subjective posteriors than those presented in a probability format, suggesting a framing effect that shifts subjective posteriors toward the likelihood of the evidence. It is important to note that these observations reflect group-level averages and do not capture individual variability in responses across participants.

Table 3 shows how subjective responses vary among individuals for evidence_1_ scenarios. It shows the percentage of participants whose subjective probabilities fell within a narrow range (±0.03) of the Bayesian prior, likelihood, or posterior. The modus is depicted in bold. In the urn tasks, some participants gave responses close to the prior, indicating that they paid more attention to it than to the likelihood. Some participants gave responses close to the posterior, indicating good calibration between attending to the prior and the evidence. In contrast, in the cab scenarios, a much larger proportion of participants gave responses near the likelihood, indicating attention to it at the expense of the prior (i.e., base-rate neglect) at the individual level (as in Bar-Hillel, 1980). Notably, few participants provided responses close to the Bayesian posterior in the cab tasks, confirming that group-level biases reflect consistent individual patterns. Additionally, a considerable number of responses in all conditions fell into the “other” category, indicating variability beyond the main trends.

**Table 3:** The percentage of subjects responding with prior probabilities, correct posterior probabilities, and likelihoods for each problem type (for evidence_1_: *n* = 1 problems only; urn = urn task, cab s = standard cab problem, cab m = modified cab problem] and the framing of problems (pro, probabilities; fre, (relative) frequencies] that are within a range of ±0.03.

	**urn_1_ pro**	**urn_1_ fre**	**cab s_1_ pro**	**cab s_1_ fre**	**cab m_1_ pro**	**cab m_1_ fre**
Prior	**0.354**	**0.292**	0.229	0.042	0.146	0.104
Posterior	0.292	0.229	0.021	0.063	0.000	0.021
Likelihood	0.250	**0.292**	**0.604**	**0.708**	**0.729**	**0.771**
Other	0.104	0.187	0.146	0.187	0.125	0.104

For example, if the prior probability was 0.20, responses ranging from 0.17 to 0.23 would be classified as prior probability responses. “Other” refers to responses outside these ranges. Statistical mode values highlighted in bold.

Inferential statistics of these data were performed on the subjective posterior log odds in two steps. First, I examined the effectiveness of manipulating the task content in the standard cab problem and its modified version. The original scenario served as a model for the standard scenario used here, and involves prior probabilities based on the relative frequencies of green and blue taxis in the virtual city. The modified scenario shifts the focus, making the prior more causally relevant, by specifying the number of drivers involved in hit-and-run incidents, rather than just using general taxi frequencies.

In Table 4, model comparison by Bayesian repeated measures ANOVA compares a null model (random effects including subject and random slopes for all repeated measures factors) with all models including content, evidence, framing, and their interactions. All models started with a uniform prior probability of 0.053. Given the data, the posterior probability of the null model dropped to effectively zero, while the posterior of the best model including only the main effects of evidence and framing increased to a posterior probability of 0.592. The log(*BF*_10_) for this best model, 8.004, strongly favored it over the null model. This suggests that evidence and framing significantly affected the posterior log odds. In contrast, content, either alone or in interaction with evidence or framing, did not appear to affect the posterior log odds.

**Table 4:** Model comparison results of Bayesian repeated measures ANOVA for the two versions of the cab scenario.

**Models**	**P(M)**	**P(M|data)**	**Log(BF_M_)**	**Log(BF_10_)**	**Error %**
**Model comparison**					
Null model (incl. subject and random slopes)	0.053	1.98 × 10^−4^	−5.637	0.000	
evidence + framing	0.053	0.592	3.264	8.004	15.381
Evidence + framing + evidence ⋇ framing	0.053	0.166	1.273	6.729	3.108
Content + evidence + framing	0.053	0.093	0.613	6.152	3.681
Content + evidence + framing + content ⋇ framing	0.053	0.040	−0.283	5.313	3.181
Content + evidence + framing + evidence ⋇ framing	0.053	0.030	−0.592	5.014	3.238
Content + evidence + framing + content ⋇ evidence	0.053	0.018	−1.104	4.515	3.978
Content + evidence + framing + content ⋇ framing + evidence ⋇ framing	0.053	0.015	−1.280	4.342	5.386
Evidence	0.053	0.013	−1.473	4.151	3.476
Framing	0.053	0.008	−1.900	3.729	2.430
Content + evidence + framing + content ⋇ evidence + content ⋇ framing	0.053	0.008	−1.935	3.694	3.547
Content + evidence + framing + content ⋇ evidence + evidence ⋇ framing	0.053	0.006	−2.205	3.426	5.919
Content + evidence + framing + content ⋇ evidence + content ⋇ framing + evidence ⋇ framing + content ⋇ evidence ⋇ framing	0.053	0.003	−2.864	2.770	7.096
Content + evidence + framing + content ⋇ evidence + content ⋇ framing + evidence ⋇ framing	0.053	0.003	−3.000	2.634	5.954
Content + evidence	0.053	0.002	−3.251	2.383	2.620
Content + framing	0.053	0.001	−3.612	2.023	3.566
Content + framing + content ⋇ framing	0.053	6.66 × 10^−4^	−4.423	1.213	3.784
Content + evidence + content ⋇ evidence	0.053	4.30 × 10^−4^	−4.861	0.775	3.424
Content	0.053	3.50 × 10^−5^	−7.368	−1.732	2.259

All models include subject, and random slopes for all repeated measures factors. The model-averaged R^2^ of this repeated measures Bayesian ANOVA reflects the proportion of variance explained by the independent variables, averaged across the models considered. The model-averaged result of R^2^ = 0.471 suggests moderate effectiveness in explaining the variability in the data, but it is quite high for a psychological study with inherent variability in human behavior. The result indicates that at least 42.7% and up to 51.3% of the variance was explained, providing a credible interval within which the plausible values of R^2^, based on the posterior distribution, are 95% likely to fall, reflecting a high degree of confidence in the proportion of variance explained.

This is most evident in the analysis of effects shown in Table 5. While both the inclusion of evidence (*BF*_*inclusion*_ = 33.219) and framing (*BF*_*inclusion*_ = 22.865) were strongly supported by the data, neither the inclusion of content (*BF*_*inclusion*_ = 0.101) nor its interactions (*BF*_*inclusion*_ < = 0.163) were supported by the data. Thus, the effects of the content manipulation in the two versions of the cab scenario on the posterior log odds were negligible, suggesting that manipulating the causal relevance of the prior probabilities was ineffective. Therefore, the data from the two versions of the cab scenario were collapsed by averaging for the following analysis.

**Table 5:** Analysis of effects by Bayesian repeated measures ANOVA for the two versions of the cab scenario.

**Effects**	***p*(inclusion)**	***p*(exclusion)**	***p*(incl|data)**	***p*(excl|data)**	**BF(inclusion)**
**Analysis of effects**					
Content	0.737	0.263	0.221	0.779	0.101
Evidence	0.737	0.263	0.989	0.011	33.219
Framing	0.737	0.263	0.985	0.015	22.865
Content ⋇ evidence	0.316	0.684	0.038	0.962	0.087
Content ⋇ framing	0.316	0.684	0.070	0.930	0.163
Evidence ⋇ framing	0.316	0.684	0.223	0.777	0.620
Content ⋇ evidence ⋇ framing	0.053	0.947	0.003	0.997	0.057

In a second step, the effectiveness of manipulating the task content from the small-world (urn) problem to the large-world scenario was tested after collapsing the data from the two content versions of the cab problem. In Table 6, model comparison by Bayesian repeated measures ANOVA compares a null model (random effects including subject and random slopes for all repeated measures factors) with all models including content, evidence, framing, and their interactions. All models started with a uniform prior probability of 0.053. Given the data, the posterior probability of the null model dropped to effectively zero, while the posterior of the best model including content, evidence, framing, and the content-by-evidence and content-by-framing interactions increased to a posterior probability of 0.372. The log(*BF*_10_) for this best model, 20.363, strongly favored it over the null model. This suggests that content, evidence, and framing significantly affected the posterior log odds. In addition, the content-by-evidence and content-by-framing interactions affected the posterior log odds.

**Table 6:** Model comparison results of Bayesian repeated measures ANOVA for the urn problem and the collapsed cab scenario.

**Models**	**P(M)**	**P(M|data)**	**Log(BF_M_)**	**Log(BF_10_)**	**Error %**
**Model comparison**					
Null model (incl. subject and random slopes)	0.053	5.34 × 10^−10^	−18.461	0.000	
Content + evidence + framing + content ⋇ evidence + content ⋇ framing	0.053	0.372	2.368	20.363	4.570
Content + evidence + framing + content ⋇ evidence	0.053	0.359	2.309	20.325	5.217
Content + evidence + framing + content ⋇ evidence + content ⋇ framing + evidence ⋇ framing	0.053	0.105	0.748	19.097	11.621
Content + evidence + framing + content ⋇ evidence + evidence ⋇ framing	0.053	0.086	0.522	18.893	5.539
Content + evidence + framing + content ⋇ evidence + content ⋇ framing + evidence ⋇ framing + content ⋇ evidence ⋇ framing	0.053	0.034	−0.462	17.964	9.729
Content + evidence + framing + content ⋇ framing	0.053	0.010	−1.721	16.730	23.613
Content + evidence + content ⋇ evidence	0.053	0.009	−1.789	16.662	6.654
Content + framing	0.053	0.007	−2.005	16.448	4.310
Content + evidence + framing	0.053	0.007	−2.021	16.432	5.730
Content + framing + content ⋇ framing	0.053	0.007	−2.044	16.410	4.162
Content + evidence + framing + evidence ⋇ framing	0.053	0.002	−3.522	14.937	5.375
Content + evidence + framing + content ⋇ framing + evidence ⋇ framing	0.053	0.002	−3.580	14.879	4.615
Content	0.053	2.13 × 10^−4^	−5.562	12.899	6.615
Content + evidence	0.053	1.76 × 10^−4^	−5.757	12.703	3.904
Framing	0.053	1.97 × 10^−8^	−14.850	3.611	5.088
Evidence + framing	0.053	1.62 × 10^−8^	−15.047	3.413	3.697
evidence + framing + evidence ⋇ framing	0.053	3.991 × 10^−9^	−16.449	2.012	3.982
Evidence	0.053	4.43 × 10^−10^	−18.647	−0.187	3.330

All models include subject, and random slopes for all repeated measures factors. The model-averaged R^2^ = 0.448 suggests moderate effectiveness in explaining the variability in the data. The result indicates that at least 40.5% and up to 48.8% of the variance was explained, providing a credible interval within which the plausible values of R^2^, based on the posterior distribution, are 95% likely to fall.

This is most evident in the analysis of effects shown in Table 7. In addition to all of the main effects, the inclusion of the content-by-evidence interaction received substantial support from the data (*BF*_*inclusion*_ = 59.219). However, the analysis of effects revealed that the inclusion of the content-by-framing interaction received only marginal support from the data (*BF*_*inclusion*_ = 2.441).

**Table 7:** Analysis of effects by Bayesian repeated measures ANOVA for the urn problem and the cab scenario.

**Effects**	**P(incl)**	**P(excl)**	**P(incl|data)**	**P(excl|data)**	**BF_incl_**
**Analysis of effects**					
Content	0.737	0.263	1.000	4.09 × 10^−8^	8.73 × 10^+6^
Evidence	0.737	0.263	0.985	0.015	23.794
Framing	0.737	0.263	0.990	0.010	36.899
Content ⋇ evidence	0.316	0.684	0.965	0.035	59.219
Content ⋇ framing	0.316	0.684	0.530	0.470	2.441
Evidence ⋇ framing	0.316	0.684	0.228	0.772	0.639
Content ⋇ evidence ⋇ framing	0.053	0.947	0.034	0.966	0.630

**Figure 1:**
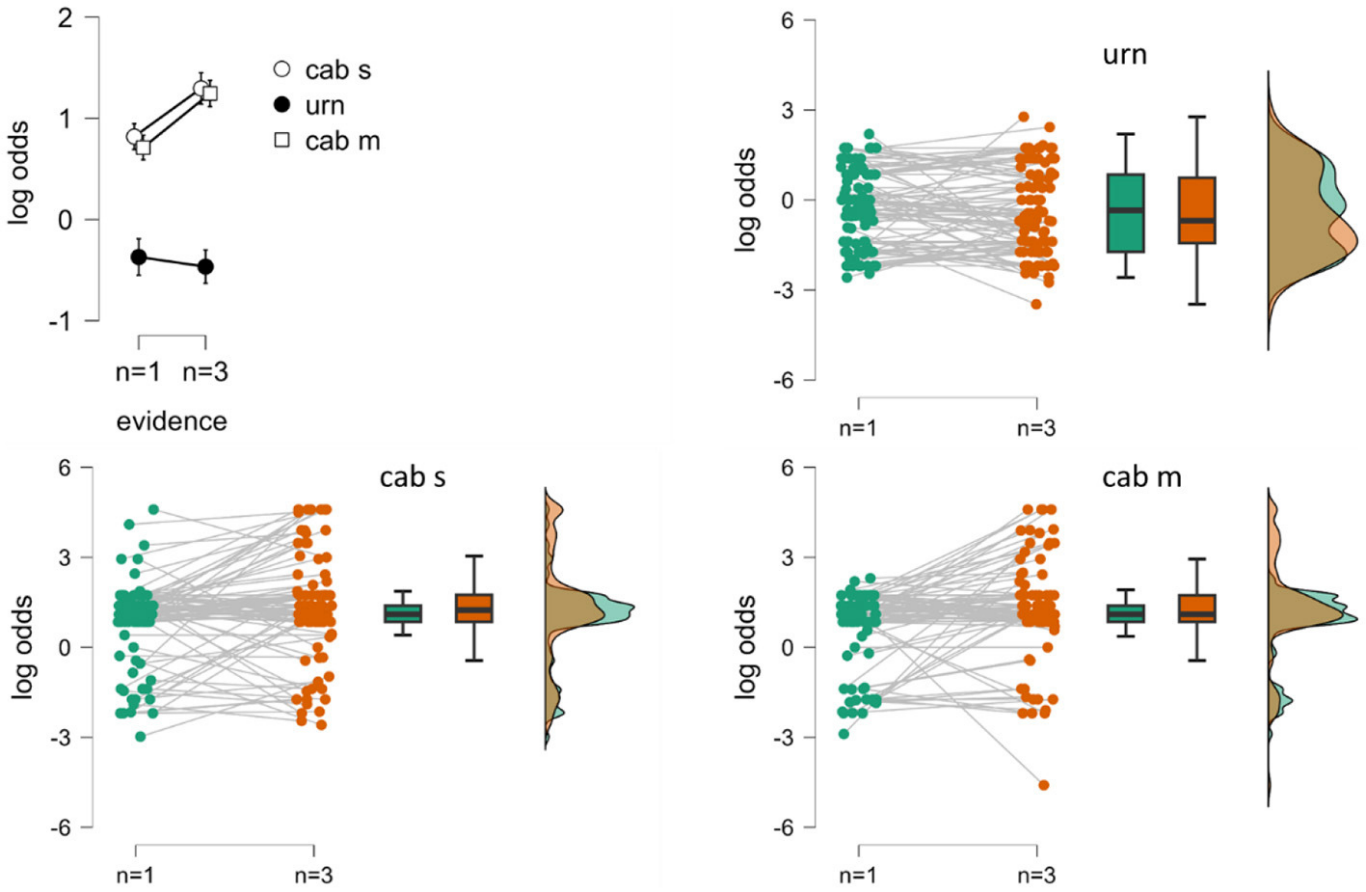
The upper left graph shows the group means (±standard errors) of subjective posterior log odds with data collapsed across the two framing conditions, separately for task content (urn = urn task, cab s = standard cab problem, cab m = modified cab problem) and level of evidence (*n* = 1, *n* = 3). The raincloud plots show the inter-individual dispersion, separately for the urn (upper right graph), the cab s (lower left graph), and the cab m (lower right graph) scenarios.

Figure 1 shows the content-by-evidence interaction, with evidence clearly affecting subjective posterior log odds in the large-world scenario (such that log odds evidence_3_ > log odds evidence_1_), but not in the small-world scenario (log odds evidence_3_ ≈ log odds evidence_1_). The effects of the content-by-evidence interaction on posterior log odds suggest that, when including paradigmatic small-world and large-world scenarios, the manipulation of task content enhanced cognitive attention to the evidence and its manipulation in the large-world scenario, apparently at the expense of cognitive attention to prior probabilities. These attentional settings may have been reversed in the small-world scenario, with greater attention to prior probabilities and less attention to evidence and its manipulation. Notably, these data provide evidence for content-dependent cognitive prioritization of either prior or evidence information in human judgment under uncertainty as a function of task content when its manipulation includes small-world and large-world scenarios.

## Discussion

This study examined how task context influences probabilistic biases by manipulating task content (small-world vs. large-world scenarios), evidence amount (single vs. multiple events), and framing (frequency vs. probability). It predicted that in large-world scenarios, such as the cab problem, cognitive attention prioritizes evidence, leading to base-rate neglect and preserved evidence sensitivity, whereas in small-world tasks, such as the urn problem, cognitive attention favors priors, leading to conservatism and reduced evidence sensitivity. Results reveal a content-by-evidence interaction: evidence affects posterior log odds in large-world scenarios more than in small-world tasks, suggesting content-dependent shifts in cognitive attention between priors and evidence as predicted. However, contrary to previous research, frequency framing does not appear to mitigate probabilistic biases by facilitating probabilistic computation.

I hypothesized that making prior probabilities causally relevant would increase their influence and reduce base-rate neglect. However, the absence of a significant difference between the two cab problem scenarios suggests that causal relevance alone may not be sufficient to mitigate base-rate neglect.

The results highlight the coexistence of probabilistic biases in human judgment, with task context playing a key role in determining which bias emerges under specific circumstances. This is consistent with previous research that has identified such biases and confirmed their validity for describing human judgment under uncertainty. However, the novel finding of the coexistence of probabilistic biases suggests that the notion of these biases is descriptive rather than explanatory. The notion of probabilistic biases illustrates what happens in human judgment under uncertainty, but does not fully explain why, when, and how these biases occur. In other words, a process model of probabilistic biases is still needed (Stengård et al., 2022).

A process model of probabilistic biases is presented in the remainder of this discussion. It formalizes the current results in the Adaptive Bayesian Cognition (ABC) model, which builds on the literature on probability weighting. This literature is further elaborated in Supplementary material C, which provides an overview of important theoretical foundations and mathematical formulations underlying probability weighting to ensure accessibility for readers who may not yet be familiar with it. By integrating probability weighting with Bayesian updating, the ABC model captures how individuals process uncertainty, adaptively weigh priors and evidence, and thus update beliefs in a variety of environments.

The theoretical foundation of the ABC model is that it provides a lens for understanding the construct of cognitive attention, and its context-sensitive dynamics between priors and evidence. A key feature of the ABC model is its ability to formalize cognitive attention with a single free parameter (denoted γ) that represents the dynamic allocation of attention between priors and evidence. This allocation of attention is modulated by the task context (denoted by the subscript Ϙ, i.e., the ancient Greek letter koppa), which determines the relative influence of priors and evidence. The ABC model thus provides a formal yet parsimonious understanding of probabilistic biases and a computational tool for exploring the mechanisms underlying human judgment under uncertainty. Derived from the data in the current study, it has a strong empirical foundation. This data-driven approach makes the ABC model well-suited for future validation, refinement, and broader application.

The mathematical formulation of the ABC model is based on Bayes' theorem expressed in log odds (denoted as *L*𝒪), as derived in Supplementary material B. In this linear-in-log-odds (LLO) version of Bayes' theorem (Equation 2), belief updating reduces to the simple addition of two terms: the log prior odds of the hypothesis (*L*𝒪_*prior*_) and the logarithm of the likelihood ratio (*LΛ*_*evidence*_):


(2) $$\begin{array}{c} {L𝒪}_{posterior}={L𝒪}_{prior}+L\Lambda_{evidence} \end{array}$$

The ABC model (see Equation 3) extends Bayesian LLO belief updating by introducing a dynamic weighting parameter (0 ≤ γ_Ϙ_ ≤ 1) that represents the competitive allocation of cognitive attention between the log prior odds and the logarithm of the likelihood ratio. The setting of this parameter value is determined by the task context (denoted by the subscript Ϙ)


(3) $$\begin{array}{c} {\hat{L𝒪}}_{posterior}=\;\gamma_{Ϙ}\times {L𝒪}_{prior}+\left(1-\gamma_{Ϙ}\right)\times L\Lambda_{evidence} \end{array}$$

where ${\hat{L𝒪}}_{posterior}$ denotes subjective posterior log odds. As you can see in Equation 3, the sum of these weights is constrained to the capacity limit of 1. This reflects the competitive nature of the weighting, where increasing attention to one source of information antagonistically reduces attention to the other. The ABC model thus imposes cognitive capacity constraints by specifying that the combined weight of the two dimensions (priors, evidence) is strictly bounded at 1. This constraint introduces a strong competition between cognitive attention to priors and evidence, respectively: Any increase in γ_Ϙ_ is necessarily associated with a decrease in 1–γ_Ϙ_, and vice versa.

Crucially, this competitive interaction is sensitive to the context. The setting of a small-world task, with its look and feel of randomness, seems to favor high γ_Ϙ_ values (γ_Ϙ_ >> 0.5), so that priors receive proportionally more cognitive attention. As a consequence, the evidence [weighted by (1 – γ_Ϙ_)], including the manipulation of its strength, receives proportionally less cognitive attention. This results in the conservatism bias, which increases with the amount of evidence. In contrast, large-world scenarios, have an impression of potentially causal variables and present seemingly convincing evidence. They seem to favor low γ_Ϙ_ values (γ_Ϙ_ < < 0.5), with the consequence that the evidence [weighted by (1 – γ_Ϙ_)], including the manipulation of its strength, receives proportionally increased cognitive attention. Because this occurs at the expense of reduced cognitive attention to priors, it results in what has historically been referred to as base-rate neglect.

**Figure 2:**
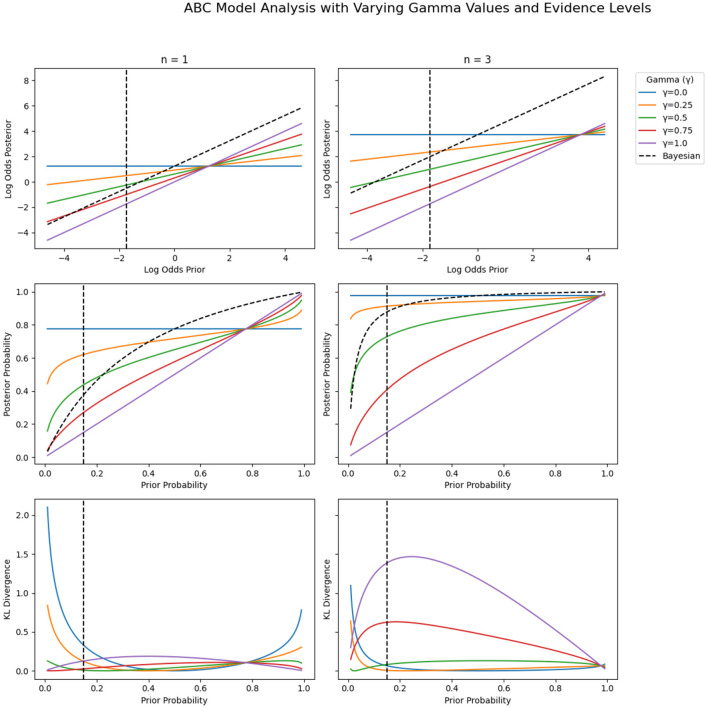
Prior-to-posterior mappings for Bayesian (dashed black line) and ABC posteriors (colored lines) separately for the two evidence conditions (for 1 (left panels) and 3 (right panels) pieces of evidence). **(Top row)** Prior-to-posterior mappings in log odds space. **(Middle row)** Prior-to-posterior mappings in probability space. **(Bottom row)** Pointwise KL divergences between Bayesian and ABC posteriors (see Supplementary Equation S.D1 for definition). Colors correspond to specific γ_Ϙ_ values in the ABC model: Blue: γ_Ϙ_ = 0.0 (mimics complete base-rate neglect), orange: γ_Ϙ_ = 0.25, green: γ_Ϙ_ = 0.5, red: γ_Ϙ_ = 0.75, and purple: γ_Ϙ_ = 1.0 (mimics extreme conservatism). The dotted vertical lines at prior=0.15 indicate the specific (average) prior used in the current study. The blue horizontal lines in the top row also indicate the two (average) levels of evidence used in the current study.

Figure 2 illustrates this. As you can see, low γ_Ϙ_ values in the ABC model cause posteriors to depend disproportionately on evidence, minimizing the influence of priors and representing hyper-flexibility when priors are low. This dynamic is consistent with cognitive tendencies annotated as base-rete neglect and jumping-to-conclusions (JTC) in psychopathological studies (So et al., 2016), and possibly with related behavioral tendencies such as distractibility or impulsivity (Kopp, 2025). Conversely, high γ_Ϙ_ values anchor posteriors to priors even in the presence of strong evidence, reflecting probabilistic biases such as conservatism and possibly related behavioral tendencies such as perseveration or rumination (Kopp, 2025).

As you can see from the pointwise KL divergences (see Supplementary material D for definition) shown in the bottom row of Figure 2, the ABC weighting effects are pervasive, as the KL divergence is almost never zero, indicating that Bayesian and ABC posteriors diverge under almost all constellations of priors and evidence, including when γ_Ϙ_ values are perfectly balanced (γ_Ϙ_ = 0.5). The pointwise KL divergences become particularly pronounced when there is a strong mismatch between priors and evidence (especially for relatively low priors and high evidence).

Taken together, these observations suggest that the highly competitive interaction between priors and evidence considered in the ABC model may account for a number of probabilistic biases observed in a variety of empirical studies. The ABC model balances the competitive interaction between priors and evidence through the weighting parameter γ_Ϙ_, which reflects context-adaptive weighting (hence the subscript Ϙ). When γ_Ϙ_ = 1, there is full reliance on the objective prior, reflecting extreme conservatism, whereas when γ_Ϙ_ = 0, there is full reliance on the evidence, reflecting extreme base-rate neglect. By modulating γ_Ϙ_, the ABC model explains how otherwise Bayesian belief updating adjusts between stability, up to full reliance on the priors with evidence neglect, and flexibility, up to full evidence sensitivity with base-rate neglect, allowing adaptation to cognitive constraints and contextual factors.

The ABC model is based on the LLO probability weighting function (PWF) of Zhang and Maloney (2012), which is detailed in Supplement Equation S.C2. An important difference between these two models is the role of evidence, because in the ABC model, the point where the PWF crosses the identity line is defined as the objective logarithm of the likelihood ratio. In the Z & M LLO model, the PWF crosses the identity line at a point called *p*_0_, which is a free parameter estimated from the data (see Supplementary Figure S.C2). Thus, the ABC model extends the basic principle of the Z & M LLO PWF, which focuses on describing subjective probabilities, to Bayesian inference. Both models use similar, though not identical, LLO transformations, but differ in their application: Following Tversky and Kahneman (1992), the Z & M model describes weighted subjective probability, while the ABC model dynamically integrates priors and likelihoods for Bayesian inference. The ABC lens suggests that probability weighting arises from the competition among probabilistic inputs (Juslin et al., 2008; Juslin, 2015), not as a general distortion of probability. In sum, the ABC model shows how subjective posterior probability emerges from the integration of priors and likelihoods through an LLO framework. This approach explains non-linear probability weighting as a byproduct of balancing priors and likelihoods, aligning subjective posterior probability with contextual demands while maintaining capacity-limited efficiency.

Other work that inspired the ABC model includes the Bayesian approach to PWF (Fennell and Baddeley, 2012), resource rational analysis (Lieder and Griffiths, 2020), and the bounded log odds (BLO) model (Zhang et al., 2020). Fennell and Baddeley (2012) proposed a Bayesian approach to PWF and showed that the combination of informative priors (based on past experience) and uninformative priors of ignorance can robustly and efficiently replicate observed PWFs, including overweighting low probabilities and underweighting high probabilities. Lieder and Griffiths's (2020) resource-rational approach integrates rational principles with cognitive constraints to model human cognition as an optimal use of limited resources. In addition, Zhang et al.'s (2020) BLO model theoretically explains the Z & M LLO PWF through bounded, dynamically adjustable internal log-odds representations that compensate for uncertainty, aligning with the resource-rational analysis by demonstrating bounded rationality as a principle that optimizes information use under cognitive constraints.

The insights provided here have both theoretical and practical implications. Theoretically, they highlight the fundamental dynamics underlying probabilistic biases such as conservatism and base-rate neglect. Practically, they provide avenues for strategies to mitigate these biases. First, frequency framing may not be sufficient to address these probabilistic biases. A more effective approach may be to encourage thinking in terms of odds and likelihood ratios. Second, one should explicitly instruct the expansion of the scope of cognitive attention by encouraging the simultaneous consideration of multiple pieces of information for integration. This simple idea leads to a generalized ABC model (Equation 4) where the limited scope of cognitive attention is modeled as a variable capacity limit, denoted by λ, with 1 ≤ λ ≤ 2 and 0 ≤ γ_Ϙ_ ≤ λ


(4) $$\begin{array}{c} {\hat{L𝒪}}_{posterior}=\;\gamma_{Ϙ}\times {L𝒪}_{prior}+\left(\lambda -\gamma_{Ϙ}\right)\times L\Lambda_{evidence} \end{array}$$

Note that the original one-parameter ABC model introduced in Equation 3 is just a case of the generalized two-parameter ABC model where λ is fixed at 1. In this model, γ_Ϙ_ reflects how priors are incorporated into belief updating, while $\frac{\lambda }{2}$ serves as a threshold for balancing priors and evidence with conservatism defined as $\gamma_{Ϙ}>\frac{\lambda }{2}$ and base-rate neglect defined as $\gamma_{Ϙ}<\;\frac{\lambda }{2}$.^1^ The rational Bayesian norm is another special case of the generalized ABC model where λ is fixed at 2 and γ_Ϙ_ is fixed at $\frac{\lambda }{2}=1$. Increasing λ through appropriate instructions to expand the range of cognitive attention minimizes the risk of neglecting available evidence (conservatism) or neglecting base rates.

Third, instructing the explicit rule to give equal weight to priors and evidence, as in the Bayesian norm, provides a structured method for appropriately balancing prior knowledge with new evidence. Such guidelines should strongly emphasize that a general principle of indifference should be to give equal weight to priors and evidence. Deviations from this principle of indifference should be considered only when the available priors are of questionable relevance to the decision problem at hand, justifying some degree of down-weighting, or when the available evidence lacks utility in terms of reliability, precision, or validity, allowing restricted down-weighting of less useful evidence in belief updating.

**Figure 3:**
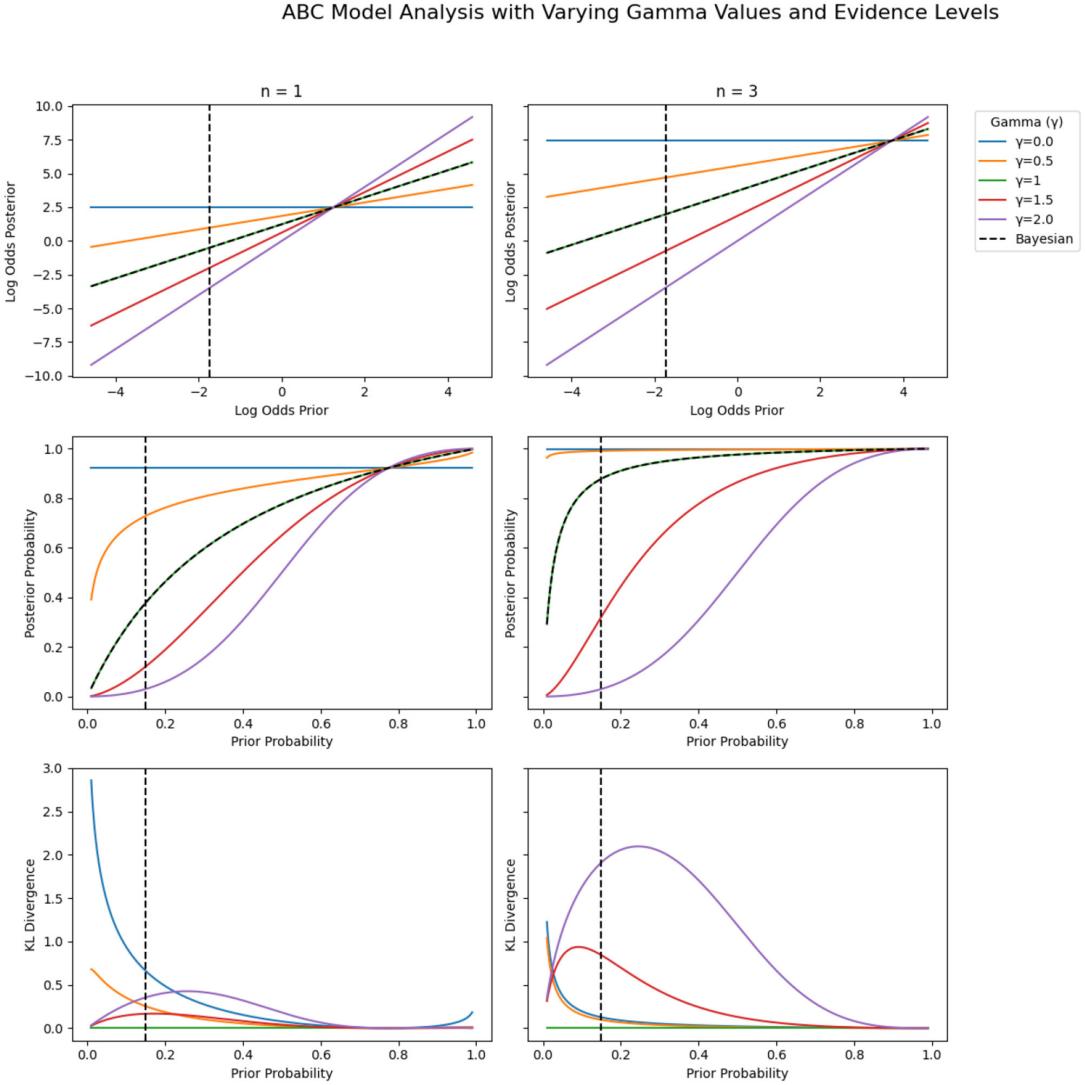
ABC prior-to-posterior transformations and KL divergences for different values of γ_Ϙ_ (0.0, 0.5, 1.0, 1.5, 2.0) for fixed λ = 2. For $\gamma_{Ϙ}=\frac{\lambda }{2}=\;1$ (green lines), the ABC posteriors are exactly equivalent to the Bayesian posteriors (hence, all pointwise KL divergences are 0). **(Top row)** Prior-to-posterior transformations in log-odds space. **(Middle row)** Prior-to-posterior transformations in probability space. The blue horizontal lines in the middle row indicate the two levels of evidence used [overweighted since here γ_Ϙ_ = 2 (blue lines)]. **(Bottom row)** Pointwise KL divergences between Bayesian and ABC posteriors (see Supplementary Equation S.D1 for definition). The dotted vertical lines at prior=0.15 indicate the specific (mean) prior probability used in the current study. Colors correspond to specific γ_Ϙ_ values in the ABC model: Blue: γ_Ϙ_ = 0.0 (mimics complete base-rate neglect), orange: γ_Ϙ_ = 0.5, green: γ_Ϙ_ = 1.0 (i.e., the Bayesian norm), red: γ_Ϙ_ = 1.5, and purple: γ_Ϙ_ = 2.0 (mimics extreme conservatism). Note the inverse S-shaped curvature of the PWF for $\gamma_{Ϙ}\leq \frac{\lambda }{2}$ and the S-shaped curvature of the PWF for $\gamma_{Ϙ}\gg \frac{\lambda }{2}$.

Figure 3 highlights the importance of this principle of indifference, which favors equal weights for priors and evidence, when cognitive attention is extended such that λ≫1. Using λ = 2 as an example, it is clear that equal weights for priors and evidence lead to normative Bayesian posteriors (being achieved when $\gamma_{Ϙ}=\frac{\lambda }{2}$). However, more extreme γ_Ϙ_ values introduce either strong hypo-flexibility (over-reliance on priors as γ_Ϙ_ approaches λ) or strong hyper-flexibility (over-reliance on evidence as γ_Ϙ_ approaches 0). Both cases lead to strongly biased posteriors, as shown in the bottom row of Figure 3. Ultimately, promoting adaptive Bayesian cognition requires tailoring strategies to support context-sensitive yet balanced weighting of priors and evidence under extended cognitive attention—ensuring flexible belief updating without sacrificing the Bayesian norm of equally weighted contributions from priors and evidence.

In summary, the generalized ABC model, defined by Equation 4, provides an adaptive framework for Bayesian inference. Distinctive features of the ABC model include its use of log odds transformations to simplify the integration of probabilistic information and its parameter flexibility, where γ_Ϙ_ and λ−γ_Ϙ_ provide fine-grained control over the influence of priors and evidence. This extends its applicability beyond traditional descriptive Bayesian models (Gigerenzer and Murray, 1987; Anderson, 1990; Oaksford and Chater, 2007; Griffiths et al., 2010, 2024) for a more nuanced contextual representation of the influence of priors and evidence.

The generalized ABC model, defined by Equation 4, can be seamlessly integrated with recent meta-analytic results. These provide empirical estimates of the weights assigned to priors and evidence in sequential urn tasks (Zhu et al., 2023), in which balls are drawn one at a time from an urn while beliefs about the underlying probabilities are repeatedly updated during the sequence of observed outcomes. From the perspective of the generalized ABC model, γ_Ϙ_ captures reliance on prior beliefs, while λ−γ_Ϙ_ adjusts the weight assigned to evidence, reflecting variations in cognitive resources and task demands. The meta-analytic results, which synthesize data from several such studies, show that individuals tend to assign high weights to priors (γ_Ϙ = *urn*_≈0.90…0.95) and comparatively lower weights to evidence (λ−γ_Ϙ = *urn*_≈0.33…0.471), based on inverse variance-weighted mean regression coefficients across studies. As expected, these results confirm a tendency toward conservative Bayesian updating, with belief updating heavily influenced by priors, reflecting the conservatism bias. From the perspective of the generalized ABC model, the analyses also indicate the need for a flexible λ parameter with estimated values of λ>1 (λ = γ_Ϙ = *urn*_+(λ−γ_Ϙ = *urn*_)≈1.23…1.42), highlighting the context sensitivity of both ABC model parameters. These meta-analytic findings support the notion that both γ_Ϙ_ and λ are dynamic and adapt to the task context. This integrated framework highlights the importance of further empirical investigation to disentangle the distinct contributions of γ_Ϙ_ and λ in different task contexts.

According to the ABC model, probabilistic reasoning is inherently Bayesian. However, it is influenced by resource constraints that create biased competition between priors and evidence. Rather than discarding Bayesian logic, individuals pay more attention to the prior or likelihood, which is influenced by cognitive limitations and contextual cues. When tasks present transparent statistical information, such as in urn problems, people tend to integrate priors and evidence more evenly. In contrast, when diagnostic details are vivid and base rates are less salient, as in cab problems, attention shifts toward the likelihood, leading to underweighting of priors. A solid body of empirical findings, including those presented here (see Table 3 in particular), shows that context determines which source dominates the competition for finite processing resources. This makes apparent deviations from Bayesian updating adaptive approximations.

The ABC model posits that heuristics emerge from a Bayesian inference system operating under resource constraints. In this model, contextual salience biases an attentional “competition” between priors and evidence rather than marking a categorical departure from normative Bayesian reasoning. Based on Gigerenzer and Gaissmaier's (2011) concept that heuristics essentially conserve effort by disregarding information, as well as the biased competition metaphor from attention research (Desimone and Duncan, 1995), the ABC model considers heuristics to be approximate Bayesian computations. In these computations, attention is drawn to the most salient cues when cognitive or contextual pressures are present. When processing capacity is limited, an attentional mechanism prioritizes either priors or likelihoods based on factors such as salience, framing, or ease of retrieval. Representativeness, for example, often dominates in cab problems at the expense of base-rate information. In contrast, in urn problems with a clearer probabilistic structure, attention is distributed more evenly. Thus, what appear as “irrational” shortcuts are reframed as adaptive, boundedly rational allocations of limited processing resources within a Bayesian architecture (see also Lieder and Griffiths, 2020).

In conclusion, within the ABC framework, heuristic reasoning (e.g., representativeness) is not qualitatively different from Bayesian reasoning (Gigerenzer and Brighton, 2009). Rather, heuristic reasoning reflects approximate Bayesian computation under resource constraints (Lieder and Griffiths, 2020). Limited cognitive resources induce biased competition between priors and evidence, which is determined by contextual salience. Thus, what appear as heuristics are simply cases where attention is preferentially directed toward certain cues, yielding adaptive yet bounded Bayesian inference (Lieder and Griffiths, 2020).

Comparison with other models reveals both overlaps and differences. The confidence-based model (Meyniel et al., 2015) also emphasizes weighting priors and evidence based on confidence, with the confidence variable similar to the role of γ_Ϙ_ in the ABC model. However, the confidence-based model treats confidence separately, whereas the ABC model uses weighted log odds transformations for simplified integration. The context model (Butz et al., 2025) extends Bayesian inference by embedding temporal and contextual dependencies in the priors. While the ABC model allows for context sensitivity through the γ_Ϙ_ term, it does not explicitly model sequential dependencies in its current form, focusing more on static belief updates. Predictive coding models (Spratling, 2017) minimize prediction errors and rely on iterative error correction. However, predictive coding emphasizes continuous feedback loops, whereas the ABC model operates with more discrete contextual updates. Resource-rational models (Lieder and Griffiths, 2020; Zhu et al., 2023), which consider cognitive constraints, overlap with the ABC model in that γ_Ϙ_ and λ−γ_Ϙ_ adjust belief updates not only to task demands but also to cognitive constraints. Kalman filter models (Kang et al., 2024), often used for sequential updating, share similarities with the ABC model in their modulation of priors and evidence, but are more specifically designed for dynamic environments. Active inference models (Friston et al., 2017), which integrate actions into the Bayesian framework, share similarities with the ABC model in terms of hierarchical inference and dynamic weighting. However, the ABC model is a purely inferential framework without the aspect of action selection. In summary, while the ABC model shares basic principles with models such as the confidence-based model, the context model, and others, its computational simplicity and precision, and its adaptability and generalizability via parameterized weighting make it a versatile and appealing tool for exploring context-dependent probabilistic cognition and belief updating within the Bayesian framework.

Metacognitive rules appear to dynamically adjust weighting parameters—perhaps reflecting how cognitive resources and contextual relevance interact with ecological sampling (Fiedler and Juslin, 2006)—thus aligning human judgment under uncertainty with both environmental demands and internal capacity constraints. The ABC model integrates related models of metacognitive learning (Binz et al., 2024), such as efficient coding (Bays, 2024) and reinforcement learning (Lebreton et al., 2019; Steinke et al., 2020; Held et al., 2024), by framing perceptual biases and cognitive control strategies as adaptive Bayesian processes. The model of online metacognitive control of decisions (Bénon et al., 2024) and the model of context-dependent adaptability of Bayesian inference in perception (Bévalot and Meyniel, 2024) complement the ABC model by framing decision making as an adaptive Bayesian process and emphasizing the context-dependent adaptability of resource allocation.

## Study limitations and future directions

The ABC model presented in this work is not a priori, but rather a posteriori. It emerged from reflection on a specific set of empirical findings. In the context of this study, the model functioned as an explanatory *post-hoc* account rather than a prescriptive, formalized computational model. The model's primary contribution is providing a transparent, mechanistically motivated framework that unifies decades of empirical phenomena, particularly those related to conservatism, base-rate neglect, and probability weighting, under the conceptual principles of γ-mediated contextual demands and λ-mediated resource constraints.

As one reviewer noted, two important limitations must be acknowledged. First, the model lacks formal parameter estimation. Second, it has not been subjected to direct, head-to-head comparisons with alternative models to assess quantitative fit. This work does not claim to offer the definitive or “best” account of the empirical phenomena in question. Rather, it represents an early stage of scientific progress: the development of a mechanistic synthesis that can serve as a generative framework for future research.

Scientific advancement in computational modeling typically proceeds in two phases: (1) an exploratory phase, in which models are developed through a posteriori theorizing about empirical observations and (2) a confirmatory phase, in which models are formalized, parameters are estimated, and rigorous model comparisons are conducted to evaluate their empirical adequacy. The present work clearly resides within the first phase. However, advancing a coherent conceptual synthesis is not merely a preliminary step; it is a precondition for meaningful formalization and exhaustive model evaluation.

Future research should aim to embed the ABC model within a broader computational program and systematically formalize its assumptions, particularly those concerning the rules of contextualized meta-learning about the utility of information across diverse environments. This refinement should include the formal parameterization of the context-dependent weighting parameter, γ, and potentially, λ. There should be a specific emphasis on deriving a priori predictions. Furthermore, as Howe et al. (2022) suggested, this effort should extend beyond pointwise probabilities to the formal consideration of full probability distributions.

One critical aspect of future work will be articulating principled, generalizable rules for determining context-dependent adjustments to γ and λ. According to the ABC model, competitive weighting arises from two interacting sources: (1) limited cognitive capacity, which necessitates prioritizing relevant inputs, and (2) environmental variability, which requires adaptive strategies for emphasizing the most reliable, precise, or valid information available. Understanding the interplay between stability (reliance on priors) and flexibility (evidence-based updating) is key to explaining how cognitive systems navigate complex and uncertain environments—a balance likely shaped by evolutionary pressures. In summary, although rigorous quantitative model evaluation is an essential next step, the present work is intended as a conceptual foundation to facilitate subsequent computational formalization and empirical testing.

The γ + (1 – γ) = 1 constraint applies to a single inference episode, during which all cognitive resources are devoted to the task at hand. In this case, paying more attention to evidence necessarily reduces attention to priors. When resources are not fully engaged, such as during lapses, shallow processing, multitasking, or divided attention, that is captured by λ < 1. This allows total weights to drop below unity. These dynamics warrant further empirical investigation.

One reviewer noted that today's posterior becomes tomorrow's prior. First, a piece of information is integrated as new evidence with weight 1–γ. Then, it is reentered as the prior with weight γ in the next update. In practice, any underweighting or overweighting of evidence carries forward and compounds over successive updates. If γ is low, new data are consistently up-weighted when first observed and down-weighted when recycled as the prior. If γ is high, the opposite occurs. This temporal cascade illustrates how slight variations in γ can result in significant dynamic effects over time when considered in discrete steps. It also suggests that incorporating γ into genuinely sequential tasks, as opposed to one-shot problems like the present ones, is a critical area for future research, possibly including the allowance of dynamic changes in γ itself.

One reviewer argued that the apparent divergence in bias—conservatism in urn problems vs. base-rate neglect in cab problems—may stem less from differences in inferential structure and more from surface-level complexity. In particular, both tasks require integrating a prior probability with new evidence; however, urn problems nest one probability within another (“proportion of urns” × “proportion of balls”), while cab problems present two independent probabilities side by side. However, if this type of complexity alone drove the effects, one would expect a uniform decline in accuracy in the more complex situation rather than systematic shifts in judgment direction. Consequently, the findings do not seem to support explanations of the observed biases based on complexity. Future work should directly and carefully manipulate complexity across both task types to isolate its impact on reasoning performance.

The study used a relative frequency format instead of the more common absolute frequency format used in Bayesian reasoning research. Absolute frequencies are known to facilitate probabilistic reasoning because they align more closely with how people naturally process information. Therefore, the current findings should be interpreted with caution when generalizing to contexts that use absolute frequency formats. Further research directly comparing these two formats would clarify their respective effects on reasoning performance.

Future research should focus on uncovering the principles of context-dependent strategy formation that govern the allocation of cognitive attention for probabilistic integration, allowing quantitative a priori predictions of weighting parameters across contexts and individuals. This includes applications to psychological disorders and neurological diseases, shedding light on individual differences (Boos et al., 2016) and clinical impairments (So et al., 2016) in human judgment under uncertainty.

Concerning individual differences, in the ABC model, γ reflects the degree to which individuals prioritize priors over new evidence. As shown in this study, urn tasks yield higher γ-values, reflecting conservative updating, while cab tasks produce lower γ-values, indicating base-rate neglect. Boos et al. (2016) applied hierarchical Bayesian modeling to estimate γ separately for each participant and task, assuming these individual γ_i_ values were drawn from a shared group-level distribution [γ_i_ ~ Beta(α, β), where α and β are weakly informed]. This approach demonstrated that γ varies systematically across task types and revealed substantial inter-individual variation. Such hierarchical modeling outperforms parameter-free or fixed-group γ models, offering superior fit and richer insight into cognitive strategies in probabilistic inference. Therefore, individualizing the ABC model through participant-specific γ_i_ parameters and appropriate hierarchical modeling is a crucial step for future research on probabilistic belief-updating behavior.

Future research should also explore the neuropsychological underpinnings of the ABC model, examining psychopathological patterns of belief updating and their neural correlates (Murphy et al., 2024). In this way, the ABC model could become a valuable framework for linking Bayesian cognition to its neural substrates (Kopp, 2008; Kopp et al., 2016; Seer et al., 2016; Lin and Garrido, 2022), providing insights into both normal and disordered brain function (Kopp, 2025). This approach has the potential to advance reverse engineering of the neural mechanisms underlying adaptive Bayesian cognition and discover the computational and neural underpinnings of the Bayesian brain (Knill and Pouget, 2004; Kolossa et al., 2015; Lin and Garrido, 2022).

## Conclusions

The ABC model frames probabilistic biases, such as base-rate neglect and conservatism, as adaptive responses to cognitive constraints and contextual demands rather than as irrational departures from Bayesian norms (Gigerenzer and Murray, 1987; Anderson, 1990; Oaksford and Chater, 2007; Griffiths et al., 2010, 2024). According to this perspective, the apparent underweighting or overweighting of priors stems from the flexible modulation of Bayesian updating. When evidence is highly salient, priors may be down-weighted, resulting in base-rate neglect. Conversely, when evidence is weak or noisy, priors may dominate, leading to conservatism. This competition for limited attentional resources is similar to cue competition in associative learning, such as Kamin's blocking, and aligns with formal associative accounts, like the Rescorla–Wagner model (Rescorla and Wagner, 1972; Juslin et al., 2008; Kopp et al., 2002). This yields non-linear probability weighting as an emergent property of adaptive, capacity-limited integration.

Within the ABC framework, two parameters govern how attention is allocated between prior information and likelihood evidence: γ, which captures contextual demands, and λ, which captures resource limitations. Specifying priors over γ and λ allows one to generate specific candidate models tailored to particular tasks or individuals, which can then be formally compared (e.g., via Bayesian model comparison). This approach enables both empirical validation and iterative refinement, as well as an explanation of variability in reasoning. What appear as opaque heuristics or biases become principled, resource-rational approximations that are tuned by task structure and individual capacity constraints.

The model predicts that shifts in framing or cognitive load will systematically alter the relative weighting of priors and evidence, thereby reproducing known bias patterns in simulations of resource-rational inference (Lieder and Griffiths, 2020). The model also suggests that decision aids can mitigate distortions by directing attention to underweighted information and ensuring the balanced integration of all information. These interventions test ABC's predictions and offer a practical means of improving judgment under uncertainty. Therefore, future research should aim to infer distributions over γ and λ in specific contexts or populations, compare ABC-based models against alternative explanations, and examine how interventions change effective attention allocation.

The ABC framework advances our understanding of human reasoning and guides the design of systems that support more accurate decision-making by recasting probabilistic biases as adaptive Bayesian approximations under cognitive constraints. Furthermore, this Bayesian process model provides a simpler explanation for cognitive biases than the dual-process models that currently dominate cognitive science's approach to biases (Kahneman, 2003). It eschews the idea of separate systems in favor of a unified, resource-rational explanation of how attentional allocation leads to biased behavior.

## Data Availability

The raw data supporting the conclusions of this article will be made available by the authors, without undue reservation.

## Footnotes

^1^The generalized ABC model (Equation 4) was already anticipated in Kopp (1988), where I introduced the power model (see Equation 23 there)

$$\begin{array}{c} {\hat{𝒪}}_{posterior}={𝒪}_{prior}^{\rho }\times \Lambda_{evidence}^{\delta } \end{array}$$

where ${\hat{𝒪}}_{posterior}$ is the subjective posterior odds. This power model assumes a multiplicative combination of odds and likelihoods, with the weights ρ and δ determining how much the prior and likelihood contribute with 0 ≤ ρ, δ ≤ 1. The exponent ρ controls for the influence of prior odds (i.e., the non-logarithmic representation of prior belief), and the exponent δ controls for the influence of lieklihoods (i.e., the non-logarithmic representation of the likelihood of the evidence). The power model defines conservatism as ρ>δ and base-rate neglect as ρ < δ. The power model is nonlinear and operates in odds space, not log-odds space (see Supplementary material B for more information). The logarithmic transformation of the power model yields ${\hat{L𝒪}}_{posterior}={\rho \times L𝒪}_{prior}+{\delta \times L\Lambda }_{evidence}$, which is equivalent to Equation 4, with ρ replacing γ_Ϙ_ and δ replacing λ−γ_Ϙ_.
