# Supplementary material for: Cognitive biases as Bayesian probability weighting in context
Source: Front Psychol. 2025 Aug 6;16:1572168. doi: 10.3389/fpsyg.2025.1572168 (PMC12366471; doi:10.3389/fpsyg.2025.1572168)
Supplement: Supplementary file 1 [file Table_1.docx]

Supplements

Contextualizing the weighting of probabilities
in adaptive Bayesian cognition

Bruno Kopp

Cognitive Neuropsychology, Department of Neurology,
Hannover Medical School, Hannover, Germany

**Correspondence:**Bruno Kopp
[kopp.bruno@mh-hannover.de](mailto:kopp.bruno@mh-hannover.de)

**Running title: Adaptive Bayesian cognition**

Supplement A:
The wording of the twelve scenarios

The twelve scenarios comprised three task contents (urn task, standard cab problem, modified cab problem) by two framings (single-case probability framing, long-run (relative) frequency framing) by two levels of evidence (*n*=1, *n*=3).

Supplement A presents the wording of all twelve scenarios, which were translated from the German language to their English equivalents with the help of DeepL.com (free version).

For simplicity of presentation, the prior probabilities (expressed as percentages, i.e., 90/10, 85/15, or 80/20 percent) are referred to throughout the scenarios as variables *p_1_* (denoting 90, 85, or 80 percent) and *p_2_* (denoting 10, 15, or 20 percent), where *p_1_* and *p_2_* sum to 100.

Similarly, the evidence likelihoods (expressed as percentages, i.e., 85/15, 80/20, 75/25, or 70/30 percent) are referred to throughout the scenarios as variables *e_1_* (denoting 85, 80, 75, or 70 percent) and *e_2_* (denoting 15, 20, 25, or 30 percent), where *e_1_* and *e_2_* sum to 100.

In the cab problems, the four evidence likelihoods were assigned to different features (here referred to as FEATURE1 and FEATURE2). Two first letters of the license plate (FEATURE1="FD" vs. FEATURE2="FE") were consistently associated with a 70/30 percent evidence likelihood. Two car-making companies (FEATURE1="Daimler-Benz" vs. FEATURE2="BMW") were consistently associated with 75/25 percent likelihood. Two colors (FEATURE1="green" vs. FEATURE2="blue") were consistently associated with 80/20 percent likelihood (as in the original cab problem). Two ad labels (FEATURE1="Coca-Cola" vs. FEATURE2="Pepsi-Cola") were consistently associated with 85/15 percent likelihood.

Finally, the two levels of evidence (*n*=1, *n*=3) are denoted here as *N* for sake of simplicity.

The title page of the booklet contained the text:

“This study forms the basis of the empirical part of my thesis. It examines human judgment and reasoning in complex problem situations. Therefore, the following problems are not easy. Take your time with each task. All the problems are different, even if they look similar. Read all the problems carefully and answer them to the best of your knowledge.

Take time to think about your answers.

Please remember that psychological studies of this kind only make sense if the subjects, i.e., you, are willing to cooperate. Please help me with your cooperation and attention in my attempt to draw valid conclusions about some human thought processes by observing your behavior as reliably as possible.

Thank you very much!”

Scenarios 1 & 2: Content=urn task, 2 levels of evidence (*n*=1 or *n*=3), framing=single case (probability)

*Content*

A statistician is conducting an experiment with two different types of urns.
Each X urn contains many balls labeled X and few balls labeled Y.
Each Y urn contains many balls labeled Y and few balls labeled X.

The statistician sets up a number of urns; then he randomly selects one urn from the set of urns.
Finally, he draws a random sample of balls from the selected urn.

*Probabilistic information*

The following information is obtained:

(i) Of the urns set up, *p_1_* % are X urns and *p_2_* % are Y urns.

(ii) The statistician draws *N* ball(s) labeled Y.

Each X urn contains *e_1_* % X balls and *e_2_* % Y balls.
Each Y urn contains *e_2_* % X balls and *e_1_* % Y balls.

Question 1: What is the probability (in percent) that the statistician has drawn the Y ball(s) from a Y urn?

This probability is ... %.

Question 2: What is the probability (in percent) that the statistician has drawn the Y ball(s) from an X urn?

This probability is ... %.

Scenarios 3 & 4: Content=urn task, 2 levels of evidence (*n*=1 or *n*=3), framing=long run (relative frequency)

*Content*

A statistician conducted a long series of experiments.
In each experiment, he used two different types of urns.
Each X urn contained many balls labeled X and few balls labeled Y. Each Y urn contained many balls labeled Y and few balls labeled X.

The statistician set up a number of urns in each experiment; then he randomly selected one urn from the set of urns. Finally, in each experiment, he drew a random sample of balls from the selected urn.

*Probabilistic information*

The following information is obtained:

(i) In each experiment of the long series of experiments, of the urns set up, *p_1_* percent are X urns and *p_2_* percent are Y urns.

(ii) In each experiment, the statistician had drawn *N* ball(s).

Each X urn contained *e_1_* percent X balls and *e_2_* percent Y balls.

Each Y urn contained *e_2_* percent X balls and *e_1_* percent Y balls.

Question 1: Consider the entire set of experiments:
In what percentage of the experiments in which the statistician drew *N* Y ball(s) did these ball(s) come from a Y urn?

In ... % of the time.

Question 2: Consider the entire set of experiments:
In what percentage of the experiments in which the statistician drew *N* Y ball(s) did these ball(s) come from an X urn?

In ... % of the time.Scenarios 5 & 6: Content=standard taxi cab, 2 levels of evidence (*n*=1 or *n*=3), framing=single case (probability)

*Content*

A taxi driver, who subsequently committed a hit-and-run, caused an accident with his taxi at night.
Two taxi companies operate in the city where the accident occurred.
All the vehicles of one company are FEATURE1 and all the vehicles of the other company are FEATURE2.

*Probabilistic information*

The following information is obtained:

(i) In the city, *p_1_* % of the taxis are FEATURE1 and *p_2_* % of the taxis are FEATURE2.

(ii) *N* witness(es) identified the fleeing taxi as FEATURE2.

The court now tests the witness(es)'s ability to distinguish between the taxi cabs under the same visual conditions that prevailed on the night of the accident.

A random sample of taxi cabs is presented to the witness(es). He(they) correctly identified each of the two taxi cabs *e_1_* % of the time. He(they) confused each of the two ads *e_2_* % of the time.

Question 1: What is the probability (in percent) that the taxi cab involved in the accident was FEATURE2?

This probability is ... %.

Question 2: What is the probability (in percent) that the taxi cab involved in the accident was FEATURE1?

This probability is ... %.

Scenarios 7 & 8: Content=standard taxi cab, 2 levels of evidence (*n*=1 or *n*=3), framing=long run (relative frequency)

*Content*

In the legal history of a city, 100 taxi drivers have been convicted of hit-and-run accidents in recent years. Two taxi companies have been operating in the city for a long time.
All the vehicles of one company are FEATURE1, all the vehicles of the other company are FEATURE1.

*Probabilistic information*

The following information is obtained:

(i) In the city, *p_1_* % of the taxis are FEATURE1 and *p_2_* % of the taxis are FEATURE2.

(ii) There were *N* accident witness(es) in each of the cases heard so far. All cases in which the witness(es) identified the taxi as a FEATURE2 were selected. In each case, the court had tested the witness(es)'s ability to distinguish between the taxi cabs.

A random sample of taxis was presented to all witnesses. All witnesses had the same ability to discriminate between the taxi cabs. The witnesses correctly identified each of the two taxi cabs *e_1_* % of the time. The witnesses confused each of the two taxi cabs *e_2_* % of the time.

Question 1: Consider the total number of trials:
In what percentage of trials was a FEATURE2 taxi cab involved in the accident where *N* witness(es) identified FEATURE2?

In ... % of the time.

Question 2: Consider the total number of trials:
In what percentage of trials was a FEATURE1 taxi cab involved in the accident where *N* witness(es) identified FEATURE2?

In ... % of the time.

Scenarios 9 & 10: Content=modified taxi cab, 2 levels of evidence (*n*=1 or *n*=3), framing=single case (probability)

*Content*

A taxi driver, who subsequently committed a hit-and-run, caused an accident with his taxi at night.
Two taxi companies operate in the city where the accident occurred.
All the vehicles of one company are FEATURE1 and all the vehicles of the other company are FEATURE2.

*Probabilistic information*

The following information is obtained:

(i) In recent years, there have been a total of 100 convictions of taxi drivers for hit and run in this city. So far, *p_1_* drivers of the FEATURE1 company and *p_2_* drivers of the FEATURE2 company have been convicted.

(ii) *N* witness(es) identified the advertisement on the fleeing taxi as FEATURE2.

The court now tests the witness(es)'s ability to distinguish between the taxi cabs under the same visual conditions that prevailed on the night of the accident.

A random sample of taxi cabs is presented to the witness(es). He(they) correctly identified each of the taxi cabs *e_1_* % of the time. He(they) confused each of the two taxi cabs *e_2_* % of the time.

Question 1: What is the probability (in percent) that the taxi cab involved in the accident was FEATURE2?

This probability is ... %.

Question 2: What is the probability (in percent) that the taxi cab involved in the accident was FEATURE1?

This probability is ... %.

Scenarios 11 & 12: Content=modified taxi cab, 2 levels of evidence (*n*=1 or *n*=3), framing=long run (relative frequency)

*Content*

In the legal history of a city, 100 taxi drivers have been convicted of hit-and-run accidents in recent years.
Two taxi companies have been operating in the city for a long time.
All the vehicles of one company are FEATURE1, all the vehicles of the other company are FEATURE2.

*Probabilistic information*

The following information is obtained:

(i) In recent years, there have been a total of 100 convictions of taxi drivers for hit and run in this city. So far, *p_1_* FEATURE1 drivers and *p_2_* FEATURE2 drivers have been convicted.

(ii) There were *N* accident witness(es) in each of the cases heard so far. All cases in which the witness(es) identified the taxi as FEATURE2 were selected. In each case, the court had tested the witness(es)'s ability to distinguish between the two taxi cabs.

A random sample of taxi cabs was presented to all witnesses. All witnesses had the same ability to discriminate between the taxi cabs. Each witness correctly identified each of the two taxi cabs *e_1_* % of the time. The witnesses confused each of the two taxi cabs *e_2_* % of the time.

Question 1: Consider the total number of trials:
In what percentage of trials was a FEATURE2 taxi cab involved in the accident where *N* witness(es) identified FEATURE2?

In ... % of the time.

Question 2: Consider the total number of trials:
In what percentage of trials was a FEATURE1 taxi cab involved in the accident where *N* witness(es) identified FEATURE2?

In ... % of the time.

Supplement B:
A gentle introduction to Bayesian theory

Bayes' theorem has already been presented in Eq. 1 of the main text in the context of a typical small-world problem. Here it is presented in a slightly more general form (where $H$ denotes a hypothesis and $E$ denotes evidence)

$P (H|E)= \frac{P(H)\times P(E|H)}{P(E)}$ (Eq. S.B1).

A common objection to the idea that human cognition follows the Bayesian norm, often equated with rationality, is that it is too computationally complex, involving multiplication and division of probabilities and an understanding of conditional probabilities. However, the computational complexity of Bayesian belief updating can be easily reduced, and thus its cognitive tractability improved, by some simple transformations.

First, Eq. S.B1 can be transformed into its odds version, which in the case of two exhaustive and mutually exclusive hypotheses ($H_{1},H_{2}$) yields

$\frac{P (H_{1}|E)}{P (H_{2}|E)}=\frac{P (H_{1})\times P(E|H_{1})}{P (H_{2})\times P(E|H_{2})}= \frac{P (H_{1})}{P (H_{2})} \times\frac{P(E|H_{1})}{P(E|H_{2})}$ (Eq. S.B2).

A mere notational simplification by using the symbol 𝒪 for the odds and *Λ* for the likelihood ratio yields

$\mathcal{O}_{posterior} =\mathcal{O}_{prior}\times\Lambda_{evidence}$ (Eq. S.B3).

Here, $\mathcal{O}_{prior}$= $\frac{P (H_{1})}{P (H_{2})}$ denotes the prior odds of hypothesis of $H_{1}$ relative to $H_{2}$, and $\Lambda_{evidence}= \frac{P(E|H_{1})}{P(E|H_{2})}$ is the likelihood ratio, expressing the evidential support that the evidence $E$ provides for $H_{1}$ relative to $H_{2}$.

Taking the logarithm transforms this into a simple additive form (log is denoted $L$ here to simplify notation)

${L\mathcal{O}}_{posterior} ={L\mathcal{O}}_{prior}+{L\Lambda}_{evidence}$ (Eq. S.B4).

Eq. S.B4 shows the linear-in-log-odds (LLO) version of Bayes' theorem, where Bayesian updating reduces to an almost trivial addition of two logarithmic quantities, i.e., log prior odds and log likelihood ratio, to yield log posterior odds. This simplicity of the LLO model of Bayesian updating provides clear computational tractability and is consistent with the idea of logarithmic scaling of mind and brain, which is also consistent with the psychophysical Weber-Fechner law (Dehaene, 2003; Juslin et al., 2011; Nieder, 2016).

Third, log posterior odds can be back-transformed to posterior probability $P (H_{1}|E)$ using the sigmoid function, which is a standard activation function in artificial neural networks used to simulate synaptic activation (Gurney, 1997)

$P (H_{1}|E)= \frac{e^{{L\mathcal{O}}_{posterior}}}{1+e^{{L\mathcal{O}}_{posterior}}}$ (Eq. S.B5).

Supplement C:
Probability weighting functions

The probability weighting function (PWF) of Tversky & Kahneman (1992; Kahneman & Tversky, 1979) has been widely used in behavioral economics (Wakker, 2010). It transforms objective probabilities into subjective decision weights that reflect how people perceive probabilities rather than their actual values. The T & K PWF is expressed as

$w\left( p \right)=\frac{p^{\gamma}}{{(p^{\gamma}+{(1-p)}^{\gamma})}^{\frac{1}{\gamma}}}$ (Eq. S.C1),

where $p$ is the objective probability, $w\left( p \right)$ is the decision weight, and $\gamma$ ($0<\gamma<1$) is the weighting parameter that controls the curvature of the PWF. The main features of the T & K PWF are overweighting of small probabilities where $w\left( p \right)>p$, meaning that individuals tend to overestimate the probability of rare events, and underweighting of large probabilities where $w\left( p \right)<p$, indicating that individuals tend to underestimate more likely events (see Figure S.C1). The empirical T & K PWF is typically inverse S-shaped, with an intersection point where the PWF crosses the identity line around $p=0.5$.


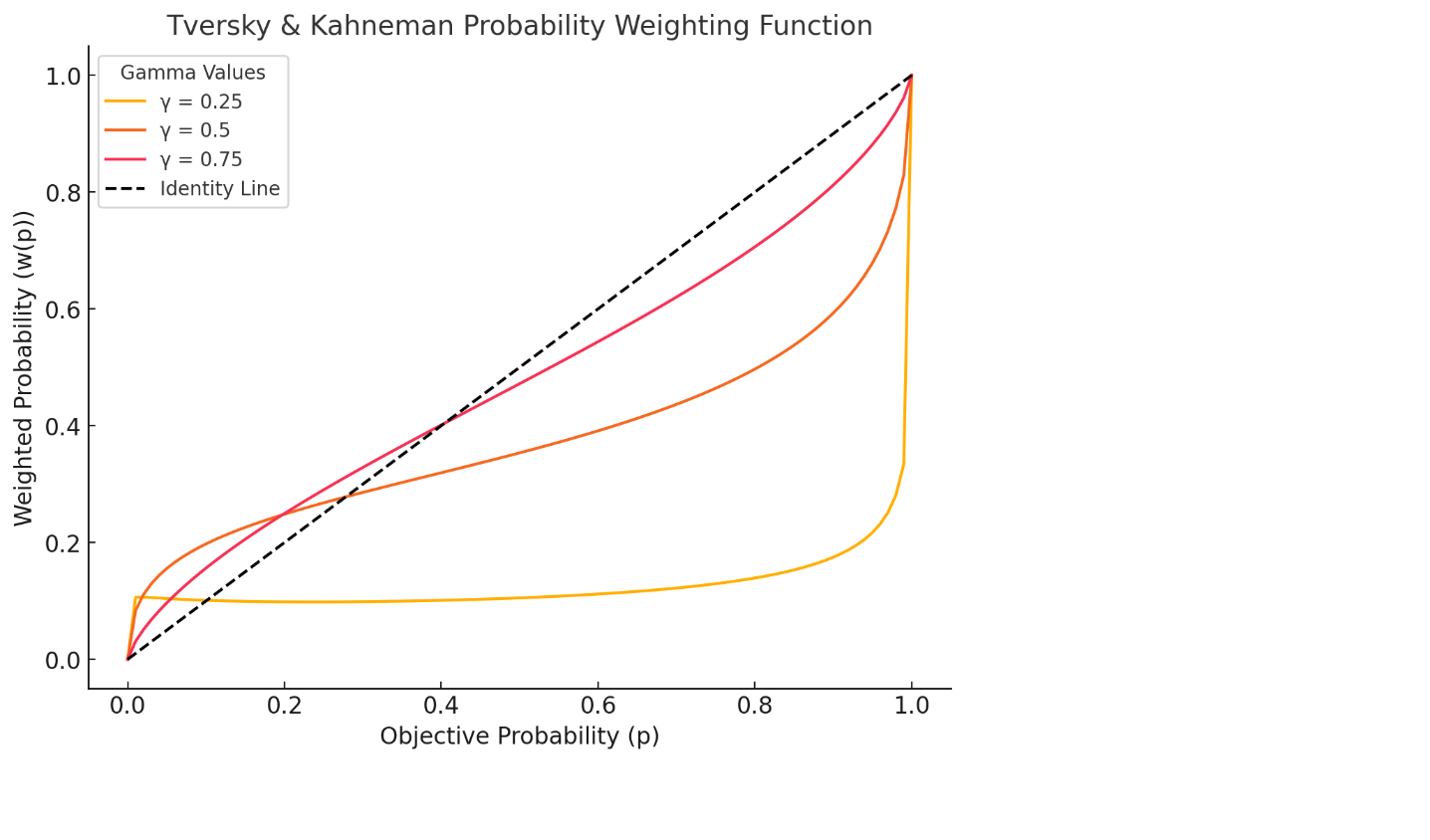
**Figure S.C1:** Plots of the Tversky & Kahneman PWF for different $\gamma$ values (0.25, 0.5, 0.75). Key observations are a pronounced overweighting of small probabilities and underweighting of large probabilities when $\gamma=0.25$; a nearly balanced inverse S-shaped PWF typical of the empirical fit when $\gamma=0.5$; and a PWF closer to the identity line, with less pronounced biases when $\gamma=0.75$. The graph includes the identity line ($w\left( p \right)$=$p$) for reference.

Zhang & Maloney (2012) significantly leveraged the insights and methodologies established by previous scholars, including Tversky & Kahneman (1992) whose work on PWFs provided the initial framework. In addition, the authors relied on the research of Karmarkar (1978), Goldstein & Einhorn (1987), and Gonzalez & Wu (1999), which informed their own investigation. Building on this line of prior research, Zhang and Maloney (2012) provided a simple linear-in-log-odds (LLO) PWF. This Z & M LLO PWF proposed that inverse S-shaped probability weighting can be more easily described in log-odds space. Here, the probability weighting results from a linear combination of the log odds of the objective probability $p$ (denoted here as $L\mathcal{O}_{p}$) and a fixed intersection point $p_{0}$. The Z & M LLO PWF has been expressed as

$L\mathcal{O}_{w\left( p \right)}=\gamma\times L\mathcal{O}_{p}+(1-\gamma)\times L\mathcal{O}_{p_{0}}$ (Eq. S.C2),

where $\gamma$ is the degree of bias from the objective probability $p$, with $\gamma$ ranging from no bias when $\gamma=1$ to full anchoring to $p_{0}$ when $\gamma=0$, where $p_{0}$ is the subjective intersection point. The Z & M LLO PWF, when converted back to probabilities, can exhibit nonlinear probability distortions, in particular inverted S-shaped PWFs (see Figure S.C2). Thus, the Z & M LLO framework showed that nonlinear, including inverse S-shaped, PWFs can arise from linear operations in log-odds space, providing a simple mathematical description of nonlinear probability weighting.


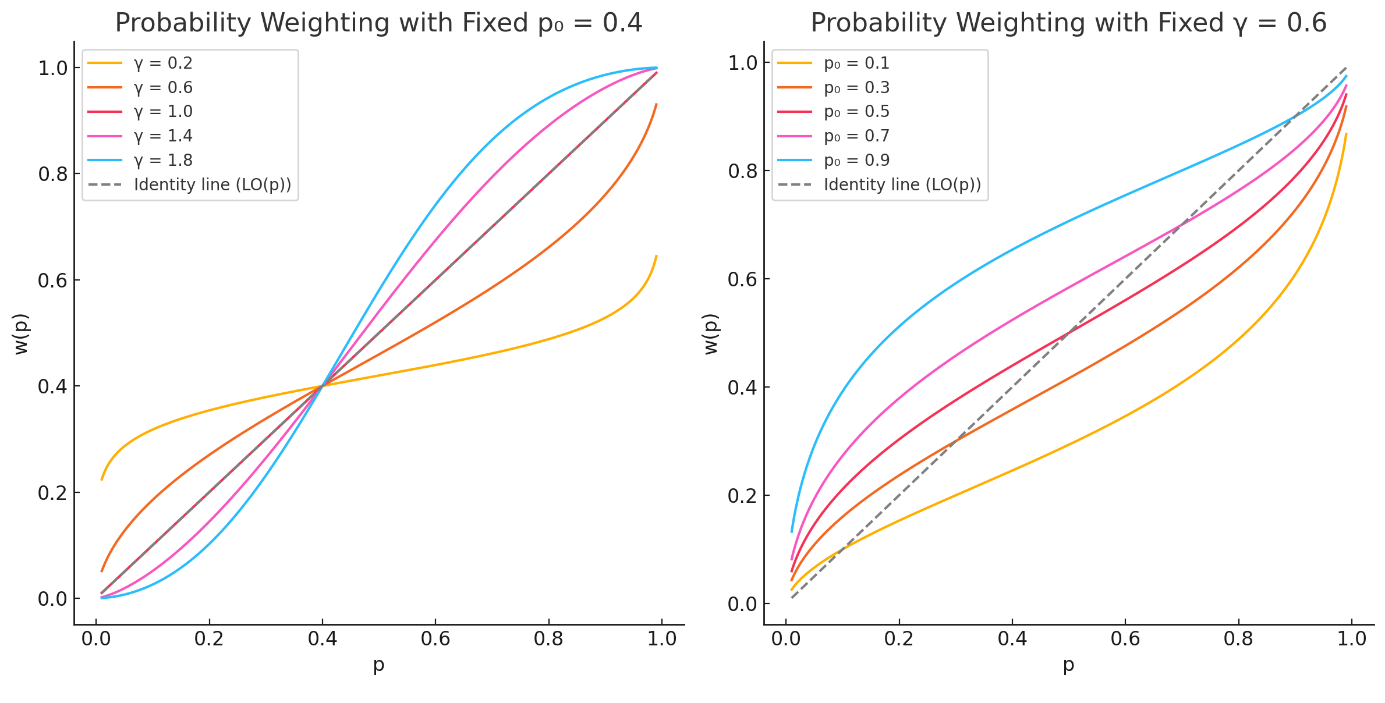
 **Figure S.C2:** Two graphs illustrating PWFs based on the Z & M LLO formalization. **Left graph**: The curvature of the PWFs varies with different values of $\gamma$ (0.2, 0.6, 1.0, 1.4, 1.8) while keeping $p_{0}=0.4$ fixed. These plots show how manipulating $\gamma$ affects the curvature of the PWFs, with inverse S-shaped PWFs when $\gamma<1$ (overweighting small probabilities, underweighting large probabilities), S-shaped PWFs when $\gamma>1$ (underweighting small probabilities, overweighting large probabilities), and PWF=identity line when $\gamma=1$. **Right graph**: The elevation of the PWFs varies with different values of $p_{0}$ (0.1, 0.3, 0.5, 0.7, 0.9) while keeping $\gamma=0.6$ fixed. You can see that the variation of $p_{0}$ shifts the position of the intersection point with the identity line in a corresponding way. Both graphs include the identity line ($w\left( p \right)$=$p$) for reference.

Supplement D:
Defining the pointwise Kullback-Leibler divergence for binary variables

The pointwise Kullback-Leibler divergences (Kullback & Leibler, 1951) were computed as follows, where $p$ denotes the posterior probabilities

$KL\left( p_{ABC}\parallel p_{Bayes} \right)=p_{ABC}\times\log\frac{p_{ABC}}{p_{Bayes}}+(1-p_{ABC})\times\log\frac{1-p_{ABC}}{1-p_{Bayes}}$ (Eq. S.D1).

**References**

Dehaene, S. (2003). The neural basis of the Weber-Fechner law: a logarithmic mental number line. *Trends in Cognitive Sciences*, *7*(4), 145-147.

Goldstein, W., & Einhorn, H. (1987). Expression theory and the preference reversal phenomena. *Psychological Review*, *94*(2), 236-254.

Gonzalez, R., & Wu, G. (1999). On the shape of the probability weighting function. *Cognitive Psychology*, *38*(1), 129-166.

Gurney, K. (1997). *An introduction to neural networks.* London, UK: UCL Press.

Juslin, P., Nilsson, H., Winman, A., & Lindskog, M. (2011). Reducing cognitive biases in probabilistic reasoning by the use of logarithm formats. *Cognition*, *120*(2), 248-267.

Kahneman, D., & Tversky, A. (1979). Prospect theory: An analysis of decision under risk. *Econometrica*, *47*(2), 363-391.

Karmarkar, U. (1978). Subjectively weighted utility: A descriptive extension of the expected utility model. *Organizational Behavior and Human Performance*, *21*(1), 61-72.

Kullback, S., & Leibler, R. (1951). On information and sufficiency. *The Annals of Mathematical Statistics, 22*(1), 79-86.

Nieder, A. (2016). The neuronal code for number. *Nature Reviews Neuroscience*, *17*(6), 366-382.

Tversky, A., & Kahneman, D. (1992). Advances in prospect theory: Cumulative representation of uncertainty. *Journal of Risk and Uncertainty*, *5*, 297-323.

Wakker, P. (2010). *Prospect theory: For risk and ambiguity*. Cambridge, UK: Cambridge University Press.

Zhang, H., & Maloney, L. (2012). Ubiquitous log odds: a common representation of probability and frequency distortion in perception, action, and cognition. *Frontiers in Neuroscience*, *6*, *1*.
